# Summer Diarrhœa in Children

**Published:** 1903-07-18

**Authors:** 


### Hospital Clinics and Medical Progress.

### SUMMER DIARRHŒA IN CHILDREN.

For purposes of treatment a very simple classification is sufficient.

1. Cases of diarrhœa and sickness, in which the general condition of the child is fairly good and the symptoms progress slowly.
2. Cases in which prostration is marked. There may be a very high or very low temperature, accordingly as the toxæmia or collapse predominates.
3. Cholera infantum, with high temperature, rice-water stools, and severe, rapidly increasing prostration.

There can be no hard and fast line drawn between these different classes, but the prognosis and treatment vary so much with the severity rather than the variety of symptoms, that this method of grouping is a serviceable one.

A typical example of the first class is as follows:—A mother brings her child with the history that for several days he has passed green, offensive stools, has been very restless and feverish, and frequently sick. On examination his skin is dry and inelastic, flesh flabby, tongue furred, pulse small and rapid. The temperature may be raised or not, but is often nearly normal. In the chest are signs of bronchitis, the abdomen is tender, rather tumid and of a doughy consistence.

The prognosis in this class of case is good. Treatment may be considered in three divisions, hygienic, dietetic, and medicinal.

*First.*—It is necessary that the child and his surroundings be kept scrupulously clean. He should have a hot bath and be wrapped in a clean blanket. The soiled napkins should be boiled for at least 15 minutes before being used again. A bottle or soother are inadmissible, and all liquids must be given with a spoon out of a cup. Plenty of fresh air is of much importance in the milder cases of summer diarrhœa. Crowding, especially collecting together several children suffering from this disease—an event which frequently happens in hospital—must be avoided. If this rule is not obeyed there is great likelihood of a severer form of the disease becoming developed and a fatal termination resulting.

*Secondly.*—Milk must be withheld altogether, and if the patient is under 18 months old, nothing given for three or four days but albumen water (2 pints in 24 hours) and plain boiled water with a little sugar in it. The child will not starve, for albumen water (made by beating up the whites of three new laid eggs in a pint of cold boiled water) is, alone, an efficient nutrient. For older children raw meat juice or gravy with toast is useful as an addition to the albumen water diet. The drugs which are most useful are castor oil, bismuth salicylate, and grey powder. On the first occasion of seeing the patient, one or two drachms of castor oil should be given, to be followed by three grains of salicylate of bismuth suspended in water every four hours (this for a child one year old). Hydrarg c̄ creta in half or one grain doses thrice daily may be substituted often with advantage when the child's general condition is good.

The second class of case is more serious and the treatment must be prompt and energetic or will fail. The symptoms come on rapidly; sometimes in a few hours an apparently healthy child is reduced to a shrunken, pale, and almost lifeless little object. The fontanelle is depressed, eyes half closed and sunken, cheeks pale, mouth dry and open, tongue furred, and abdomen retracted. Occasionally there are convulsions. The pulse is usually rapid, irregular, and small. The stools are frequent, thin, yellow and flatulent or slimy and blood-stained and very irritating to the skin around the anus. Either vomiting or diarrhœa may predominate or they may be equally frequent, so that the child's body is depleted of fluid in a very short time. The urine is scanty and highly coloured and albuminous or entirely suppressed. In those cases which terminate fatally it is the almost invariable rule to find on post-mortem examination acute nephritis, and this must be borne in mind when discussing the advantages of various forms of drug treatment.[1]

In treating these severe cases of diarrhœa and sickness the same hygienic and dietetic rules apply as in the milder forms. The child should have as much as it wants to drink, of water—not milk, and this for several reasons. Milk is a food and not a drink for the child, it cannot digest the milk and the curds formed are excellent media in which the micro-organisms manufacture the toxins by which the child is being poisoned. The most important therapeutic measure is rectal, and in cases where there is much vomiting gastric, lavage. In all cases of severe diarrhœa the rectum should be washed out, but the most marked beneficial effect will be seen in those cases of collapse and depletion which form the more serious section of this class. The details of the method which experience has shown to be most satisfactory in every way are as follows:—Take off all the child's clothes, then wrap the upper part of his body in a bath towel, let the buttocks lie in a shallow bath, bed slipper, or rubber appliance so that the water running from the rectum may not soil more material than can be helped. Take a douche-can or glass funnel, connect it with a soft rubber catheter (Jacques No. 10) having an extra hole cut on the opposite side to the usual terminal one, and about 1 inch higher up.

Put the end of the catheter into the child's rectum about 2 inches, fill up the funnel or douche-can with salt and water, a drachm to the pint, and let it run slowly through the catheter. The level of the fluid in the reservoir should be 2 feet above the rectum of the child. Continue to fill up the can, and as the fluid runs in at intervals, press the catheter higher into the bowel. The rectum will empty itself frequently by the side of the catheter. Do not stop this lavage till the fluid returns clear and inoffensive. Often two or three quarts of saline solution is hardly sufficient to secure this result. Weak Condy and water may be substituted for the saline solution.

The great advantage of using a large reservoir is that an assistant to hold the jug is unnecessary. The child, after the first few minutes of this treatment, becomes quiet and goes to sleep.

Quite an appreciable quantity of the liquid is absorbed, and aids in replenishing with fluid the dried and thirsty tissues. If there is much pyrexia cold water should be used, and if the temperature is sub-normal water at 100° to 110° Fahr. To wash out the stomach a similar catheter or No. 8–10 œsophageal tube should be passed through the nose into the stomach of the patient, the fluid going in from the funnel or douche-can being vomited through the mouth at intervals as the lavage proceeds.

The nasal tube should not be withdrawn till the vomit is clear. Just before removing this tube 10 or 15 grains of bismuth carbonate may be run into the stomach if symptoms of gastritis have been marked. Drugs are of comparatively little curative value in this class of case. Bismuth salicylate and opium, the latter in the form of half-minim doses of the tincture given after every other loose motion, may be invaluable if the diarrhœa is incessant, and the straining and distress very great. But lavage and a little stimulant associated with a completely changed diet containing no milk does more good than antiseptic drugs.

The collapse and prostration must be treated by adding 10 or 15 drops of brandy or tincture of musk to the albumen water every two hours, and placing the child in a hot bath and keeping it wrapped in a warm clean blanket. If the buttocks are much excoriated they should be thoroughly washed with soap and water, dried, and then swabbed with calamine lotion or dusted with a powder containing a drachm each of zinc oxide and bismuth carbonate added to half an ounce of violet powder.

Cholera infantum needs little description, it has but to be seen to be diagnosed. The child is collapsed, pallid, and bluish, eyes sunken and dull, tongue red and protruded from the partly open mouth. The patient is cold, apathetic or unconscious, and moribund. The temperature in the rectum often rises to 106°, or even higher, and there may be delirium or convulsions. The stools are large, of rice-water consistence and colour, and acid. The urine is usually suppressed, but there is no evidence of acute nephritis in the fatal cases. Treatment consists in first emptying and washing out the stomach and rectum, the latter with cold water, iced if necessary. This acts in several beneficial ways; it reduces the temperature, removes much of the poison, and supplies fluid to the tissues. Some stimulant, such as brandy, must be given hypodermically, or, well diluted, by the nasal tube. Attempts must be made to neutralise the effect of the poison (which is probably allied to muscarin[2]) by the administration of atropine $\frac{1}{500}$ gr., and morphine $\frac{1}{50}$ gr., which dose can be repeated by mouth two or three times at intervals of two hours. The extremities must be kept warm, and as the collapse passes off albumen water and brandy must be administered by mouth, but no milk for at least the first week of convalescence.

If the child is very shrunken, supply fluid by subcutaneous injection of saline solution containing five per cent. dextrose as a nutrient addition.[3]

To sum up. The chief points in treating all forms of summer diarrhœa are:

1. Cleanliness, external and internal, secured (*a*) by hot baths and clean, well-ventilated clothes; (*b*) gastric and rectal lavage.

2. The supply of much fluid to make up for that lost by the diarrhœa.

3. The giving of food the easiest to digest, the most difficult for the organism forming the poison to live upon.

[1] Diseases of Infancy and Childhood. (Holt. 1903. Page 360.)
[2] Brunton, "Medico-Chirurgical Transactions," vol. lxxvi.
[3] Hospital, May 16, 1903, "Some Points in Invalid Feeding."

